# Evaluating the Impact of Programmatic Mass Drug Administration for Malaria in Zambia Using Routine Incidence Data

**DOI:** 10.1093/infdis/jiaa434

**Published:** 2020-07-21

**Authors:** Maya Fraser, John M Miller, Kafula Silumbe, Michael Hainsworth, Mutinta Mudenda, Busiku Hamainza, Hawela Moonga, Elizabeth Chizema Kawesha, Laina D Mercer, Adam Bennett, Kammerle Schneider, Hannah C Slater, Thomas P Eisele, Caterina Guinovart

**Affiliations:** 1 PATH Malaria Control and Elimination Partnership in Africa (MACEPA), Seattle, Washington, USA; 2 PATH MACEPA, Lusaka, Zambia; 3 National Malaria Elimination Centre, Zambia Ministry of Health, Lusaka, Zambia; 4 Center for Applied Malaria Research and Evaluation, Department of Tropical Medicine, Tulane University School of Public Health and Tropical Medicine, New Orleans, Louisiana, USA; 5 University of California San Francisco, San Francisco, California, USA; 6 Barcelona Institute for Global Health (ISGlobal), Hospital Clínic-Universitat de Barcelona, Barcelona, Spain

**Keywords:** impact evaluation, malaria, mass drug administration, routine data

## Abstract

**Background:**

In 2016, the Zambian National Malaria Elimination Centre started programmatic mass drug administration (pMDA) campaigns with dihydroartemisinin-piperaquine as a malaria elimination tool in Southern Province. Two rounds were administered, 2 months apart (coverage 70% and 57%, respectively). We evaluated the impact of 1 year of pMDA on malaria incidence using routine data.

**Methods:**

We conducted an interrupted time series with comparison group analysis on monthly incidence data collected at the health facility catchment area (HFCA) level, with a negative binomial model using generalized estimating equations. Programmatic mass drug administration was conducted in HFCAs with greater than 50 cases/1000 people per year. Ten HFCAs with incidence rates marginally above this threshold (pMDA group) were compared with 20 HFCAs marginally below (comparison group).

**Results:**

The pMDA HFCAs saw a 46% greater decrease in incidence at the time of intervention than the comparison areas (incidence rate ratio = 0.536; confidence interval = 0.337–0.852); however, incidence increased toward the end of the season. No HFCAs saw a transmission interruption.

**Conclusions:**

Programmatic mass drug administration, implemented during 1 year with imperfect coverage in low transmission areas with suboptimal vector control coverage, significantly reduced incidence. However, elimination will require additional tools. Routine data are important resources for programmatic impact evaluations and should be considered for future analyses.

## Teaser

This study evaluated 1 year of programmatic mass drug administration (pMDA) on malaria incidence in southern Zambia and found that areas where pMDA was conducted saw a 46% greater decrease in incidence at the time of intervention than comparison areas.

Over the last decade, there has been a renewed push toward malaria elimination. Although some progress has been made, if current trends continue, we will fall short of the World Health Organization (WHO) goal of a 90% reduction in incidence worldwide by 2030. In the last 2 years, many countries saw plateaus, or even increases, in their number of malaria cases [1].

One tool being discussed as a potential accelerator to burden reduction on the path to elimination is mass drug administration (MDA), where the entire eligible population of an area are given antimalarials at the same time, once or multiple times before or during the high malaria transmission season [2]. The aim is to decrease the human parasite reservoir and provide prophylaxis into the malaria season. Although MDA was used historically, concerns about drug resistance and its short-term impact led to MDA not being recommended for many years [3]. It is only in the last half decade that MDA has been re-examined for use in malaria elimination. In 2015, an extensive review of MDA for malaria concluded that MDA should be considered for malaria control and elimination in low transmission settings [4]. Later that year, the WHO recommended that MDA could be used for *Plasmodium falciparum* malaria in settings approaching elimination that had “good access to treatment, effective implementation of vector control and surveillance, and a minimal risk of re-introduction of infection,” as well as in places in the Mekong region facing multidrug resistance. Particularly relevant to malaria control in the age of coronavirus disease 2019, MDA is also recommended as an initial part of malaria epidemic control and in emergency situations where normal malaria control activities are impossible. The WHO also urged further research into implementation, community acceptance/sensitization, and impact evaluation [5].

Starting in 2014 and continuing through early 2016, the area of Southern Province bordering Lake Kariba in Zambia was the site of a large community cluster-randomized controlled trial (CRCT) that tested 2 years (4 rounds) of MDA and focal MDA (giving antimalarials to households where any member tested positive via a rapid diagnostic test [RDT]) [6]. In the first year of the intervention, one round was conducted in December 2014 and a second round in February–March 2015. In the low incidence strata (≤10% infection prevalence), there was a 41% greater reduction (incidence rate ratio [IRR] = 0.59; 95% confidence interval [CI] = 0.44–0.78) in the incidence of passively detected malaria cases in the MDA group compared with the control group during the malaria season after the first year of MDA. In the high incidence strata (>10% infection prevalence), there was no significant effect on case incidence. A follow-up survey in 2015 suggested that the coverage had been high, with 88.1% and 72% coverage in rounds 1 and 2.

Subsequently, the National Malaria Elimination Centre (NMEC) within the Ministry of Health in Zambia began programmatic MDA (pMDA)—MDA that is part of the government malaria control strategy and not part of a research study—in Southern Province starting in late 2016. This current analysis evaluates the effect of this initial implementation, with the goal of testing the performance of MDA under programmatic—“real world”—conditions. In this study, we present an assessment of the short-term impact of pMDA on the incidence of confirmed malaria cases, using routine data collected at health facilities and by community health workers.

## METHODS

### Setting

Southern Province is 1 of 2 low-transmission provinces within Zambia, with a prevalence of *P falciparum* infection by blood smear of 0.6% [7], and an overall incidence of 26.7 cases/1000 per year in 2015, the year before the pMDA intervention [8]. Transmission is perennial, with a high season between December and June and a peak in March–April. The national strategy calls for case management with RDTs and treatment with artemisinin-based combination therapy, with first-line drug artemether lumefantrine. Facility-based care is augmented through an extensive system of community health workers (CHWs) who provide malaria information, testing via RDTs, and treatment, as well as malaria case investigations with reactive case detection where possible. In the 2015 Malaria Indicator Survey, 76% of households in Southern Province reported owning at least 1 long-lasting insecticide-treated net (LLIN), with 57% of household members reporting sleeping under a net the previous night [8]. Because the last mass distribution of LLINs before pMDA occurred in late 2014, coverage was lower at the point of intervention than it was at the time of the survey. Some health facility catchment areas (HFCAs) also received indoor residual spraying (IRS) (see Study Design).

### Intervention

The first round of pMDA was conducted in November 2016, just before the start of the malaria season, in all HFCAs in Southern Province with incidence >50 cases/1000 per year (May 2015–April 2016). The second round was targeted for 30 days after the first round, but flooding delayed it to between 56 and 67 days after the first round. Two-person campaign teams composed of a CHW and an adherence officer visited each household within the HFCA to conduct the pMDA. All household members were eligible for treatment with dihydroartemisinin-piperaquine (DHAP), excluding children under 3 months, women in their first trimester of pregnancy, and those treated with an antimalarial in the last 7 days. The CHW observed household members take the first dose, and the adherence officer followed up on the third day to check the blister packs to see whether the second dose had been taken and observe the third. The reported adherence was 97% for round 1 and 96% for round 2. The overall programmatic coverage $\left(\frac{number\;reported\;treated\;by\;MDA\;teams}{number\;t\arg eted}\right)$ was 70% in round 1 and 57% in round 2, using HFCA population denominators provided by the district as the number targeted. Coverage was likely lower in the second round due to floods that limited access to some villages.

### Study Design

**Figure 1.**
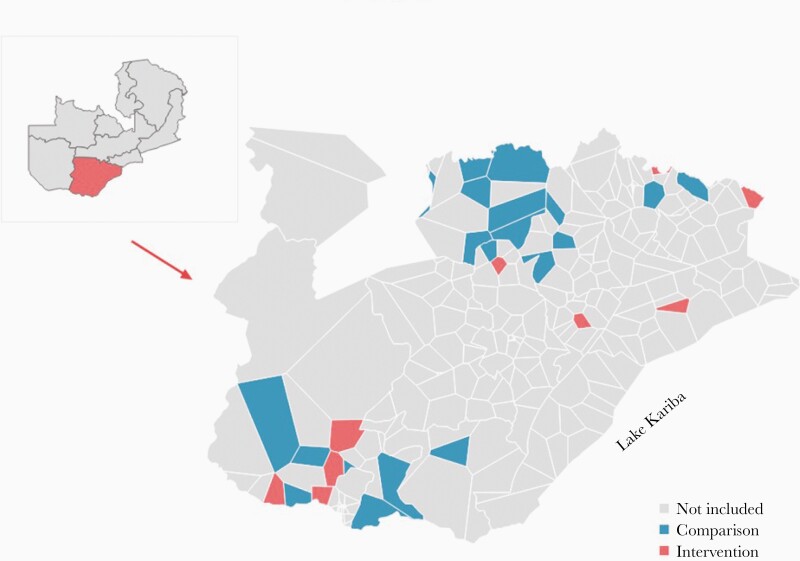
Health facility catchment areas included in the analysis, Southern Province.

A quasi-experimental design with nonrandom groups was used to assess whether the change in incidence before and after the intervention was different in an intervention group and a comparison group. These groups were chosen through a design similar to a regression discontinuity, where treatment is determined based on a cutpoint on a continuous metric—in this case, incidence of malaria cases. The HFCAs were assigned by the NMEC to receive pMDA based on a threshold of having ≥50 cases/1000 per year using data from the previous year. For our analysis, we selected pMDA HFCAs that had rates marginally above this threshold, and comparison HFCAs that had rates marginally below, to ensure the 2 groups were as comparable as possible. The pMDA group consisted of 10 HFCAs that had an incidence of 50–70 cases/1000 per year, and the comparison consisted of 20 HFCAs that had an incidence of 30 to <50 cases/1000 per year. Of these, 8 and 7 HFCAs received IRS in late 2016, respectively. The total population in these HFCAs was an estimated 126 000 in 2016 (see Data). Due to slight differences in incidence calculation from the NMEC, when we recalculated incidence, we found that some catchments fell on the opposite side of the cutoff from what their treatment status would suggest, resulting in more comparable groups than anticipated. Figure 1 shows the map of the pMDA and comparison HFCAs, and Supplementary Figure 1 shows malaria incidence before and after the intervention by individual HFCA.

All selected HFCAs met the following criteria: (1) its facility was open and submitting weekly reports into the NMEC malaria rapid reporting system in District Health Information System 2 (DHIS2) since November 2013; (2) it submitted at least 85% of expected reports from November 2015 to May 2018; (3) it had at least 1 CHW who performed malaria reactive case detection and submitted monthly reports; and (4) it was not a part of the MDA CRCT previously conducted in Southern Province.

### Data

Monthly confirmed malaria incidence data at the HFCA level from the DHIS2 malaria rapid reporting system were used as the outcome variable in the impact analysis. These data, and other testing, case investigation, and reactive case detection data elements, have been collected since 2013 by facilities and CHWs and are the subject of yearly data quality audits comparing values in the DHIS2 data system with those in the log books at the facility (source documents). On average, the facilities included in the study had accuracies of 78%, 85%, and 89% in 2014, 2016, and 2017, respectively, where accuracy is given as $\frac{\left(source-|source-system|\right)*100}{source}$ or as 0% for a negative value. Errors found during the audits were corrected in the rapid reporting system.

To estimate the population denominators used to calculate the incidence rate by HFCA, district-level population estimates (from the Central Statistical Office of Zambia) were distributed to HFCAs proportional to the number of outpatient attendees in the last year at each health facility.

Rainfall estimates and eMODIS Normalized Difference Vegetation Index were downloaded from the Famine Early Warning Systems Network and aggregated across each month [9]. Temperature data were downloaded from the LAADS DAAC (Level-1 and Atmosphere Archive & Distribution System Distributed Active Archive Center) portal of the NASA website [10].

The IRS program data were compiled by Ministry of Health and PATH staff once received from district spray managers. We calculated the percentage population covered by IRS using programmatic estimates of the number of people living in sprayed households divided by the population in the HFCA. Long-lasting insecticide-treated net distribution campaign data by district were obtained from the NMEC. A proxy for LLIN ownership was calculated by taking district-level net distribution data and distributing them proportionally by population among HFCAs. We accounted for LLIN degradation over time by applying a decay function using the decay rate from Bhatt et al [11]. Information about the pMDA campaigns came from the NMEC as well.

### Data Analysis

To test whether the change in incidence was different in the intervention and comparison groups, we used an interrupted times series with comparison group (ITSc) [12], a recommended analytical strategy for impact evaluation analyses using routine data [13]. An ITSc allows us to investigate both whether there was an immediate “level” change (equivalent to a change in intercept) at the time of intervention and whether the overall “trend” changes after the intervention compared with the trend before the intervention (equivalent to a change in slope). We used 1 interruption point, coinciding with the first round of pMDA. We included 4 variables to capture this in the model (Supplementary Table 1): months since the beginning of the study (continuous), months since the start of the intervention (continuous), before or after intervention (binary), intervention or comparison group (binary). In addition, the analysis included interaction terms between several of these variables: intervention group and months since the beginning of the study, intervention group and before or after intervention, and intervention group and months since the start of the intervention. It is these last 2 interaction variables that let us know, respectively, whether the level change and the trend change in the pMDA group are different than those in the comparison group.

To account for epidemiological and geographical differences between groups, we included the following covariates: (1) environmental factors - elevation (continuous), smoothed rainfall (weighted average of past 3 months; continuous), lagged Normalized Difference Vegetation Index (2-month lag; continuous), and month of year (binary); (2) other malaria control interventions - estimated LLINs per 1000 population (continuous) and estimated IRS coverage (continuous); (3) malaria case management and care seeking - percentage of total malaria cases reported by CHWs, number of tests per malaria index case during reactive case detection (continuous), and total nonmalaria health facility visits to control for external changes in care-seeking such as flooding or facility closure (continuous). We also included the previous month’s cases as a variable to control for autocorrelation.

We used a generalized estimating equations-based negative binomial model (GEE). GEEs are marginal models that estimate a population–average response, as opposed to conditional models that are subject-specific. GEEs take into account repeated measurements from the same subject (or in this case, HFCA) and are robust to misspecified correlation structures when robust standard errors are used. We used an autoregressive correlation structure of lag 1, which assumes that points are correlated in time, and an offset term of the log of the population associated with each HFCA, which accounts for the different catchment populations of the HFCAs.

We used malaria incidence data from the 3 transmission seasons before the introduction of pMDA (November 2013 to November 2016) to establish a baseline transmission level and 1 year after (December 2016 to November 2017) to assess the impact of pMDA. In an ideal setting, a longer outcome period would be available, but after 1 year, HFCAs received pMDA based upon the incidence from the first year of the intervention, making an unbiased longer follow-up period untenable.

## RESULTS

Descriptive summaries of all covariate values by pMDA and comparison group are presented in Supplementary Table 2. There was no significant difference between baseline incidence of malaria cases in the pMDA and comparison groups (IRR = 1.392, *P* = .395, 95% CI = 0.650–2.894) (Table 1), although the value is consistent with the intervention group having a slightly higher starting incidence.

**Table 1.** Results of ITSc Regression Analysis

Variable^a^	Incidence Risk Ratio	*P* Value	95% Confidence Interval
Pre/postintervention ^x^ intervention group (level change)	0.536	.008	0.337–0.852^b^
Months since intervention^ x^ intervention group (slope change)	1.029	.494	0.947–1.119
Months since beginning of study x intervention	0.997	.758	0.978–1.017
Months since beginning of study	1.000	.997	0.988–1.012
Months since intervention	1.049	.078	0.995–1.107
Pre/postintervention (binary)	0.601	.010	0.408–0.885^b^
Intervention group (binary)	1.392	.395	0.650–2.894
Usable nets per person	0.302	.000	0.202–0.452^b^
Total facility visits	6.890	.000	2.942–16.135
Percent of malaria cases treated by CHW	1.300	.040	1.012–1.67^b^
Average number of reactive tests per index case	0.984	.000	0.975–0.993^b^
Percentage of population covered by IRS (people protected/estimated HFCA population)	0.831	.187	0.631–1.094
Log (previous month cases +1)	1.366	.000	1.258–1.483^b^
Elevation (m)	0.998	.007	0.997–0.999^b^
Rainfall smoothed (mm)	0.999	.933	0.987–1.012
NDVI lagged	1.007	.258	0.995–1.018

Abbreviations: CHW, community health worker; HFCA, health facility catchment area; IRS, indoor residual spraying; ITSc, interrupted times series with comparison group; NDVI, normalized difference vegetation index.

^a^Categorical variables for month of year included but not shown.

^b^ *P* ≤ .05.

There was a significant level change in incidence of 40% at the time of the intervention across the intervention and the comparison group (IRR = 0.601, *P* = .01, 95% CI = 0.408–0.885). The pMDA areas saw an additional 46% level change (IRR = 0.536, *P* = .008, 95% CI = 0.337–0.852), leading to an overall level change in incidence of 68% in the pMDA group. The trend change (change in the slope after the intervention compared with preintervention) in the pMDA group was not significantly different from that in the comparison group (IRR = 1.029, *P* = .494, 95% CI = 0.947–1.119), indicating no change in the trajectory of malaria incidence due to the intervention.

Usable nets per person was also a significant and sizeable predictor of malaria incidence (IRR = 0.302, *P* < .001, 95% CI = 0.202–0.452), although IRS was insignificant. There was a positive association between the percentage of cases being treated by a CHW and malaria incidence (IRR = 1.300, *P* = .040, 95% CI = 1.023–1.67), potentially indicating that having access to CHWs leads to a higher detection rate for cases or that CHWs in higher transmission areas are more active. In addition, more follow-up cases per index case in case investigation was significantly associated with fewer incident malaria cases (IRR = 0.984, *P* < .001, 95% CI = 0.975–0.993).

**Figure 2.**
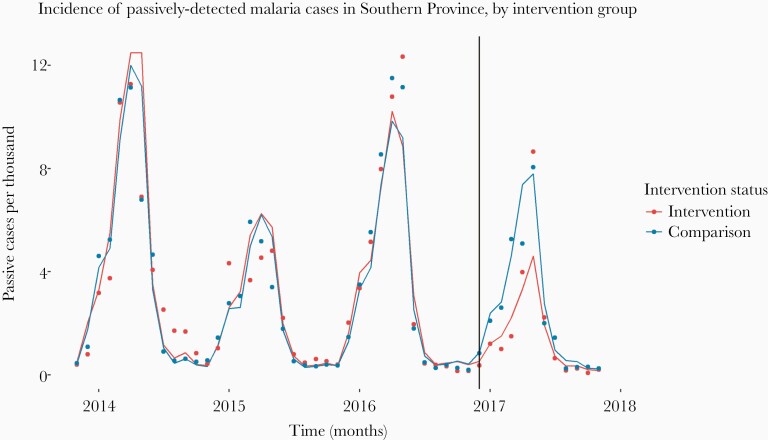
Aggregated incidence across intervention (programmatic mass drug administration [pMDA]) and comparison groups with model fits: points are observed values, and lines are fitted values from the generalized estimating equations model. The vertical line indicates the timing of the first round of pMDA. The model fit varied by health facility, with the fit notably poor in some health facilities in 2016.

Figure 2 shows the monthly incidence of passively detected malaria cases in the pMDA and comparison groups and the fit of the GEE model summarized across all HFCAs. The model captures the lower incidence in the first few months after the intervention in the pMDA HFCAs, but it fails to completely capture the seasonal peak in incidence at 4 months after the first round of pMDA. By the end of the 2016–2017 transmission season, incidence had begun to converge once again between the 2 groups.

**Figure 3.**
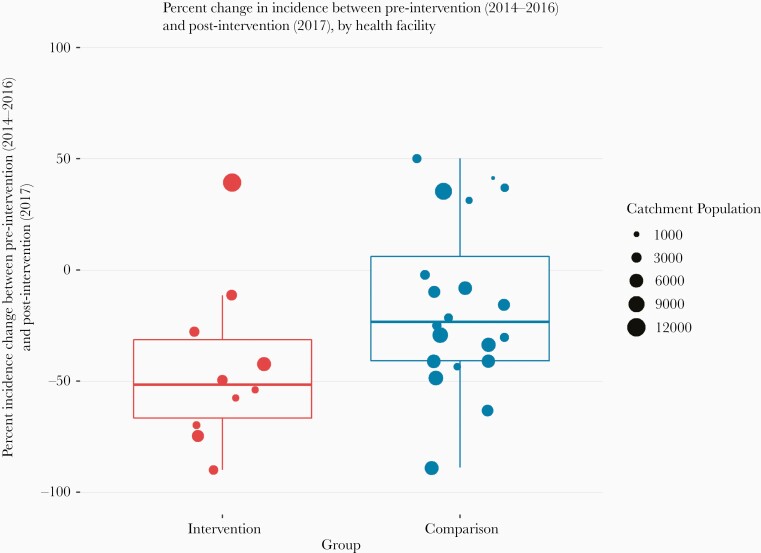
Unadjusted percentage change between baseline and postintervention period by health facility for the programmatic mass drug administration and comparison groups.

**Figure 4.**
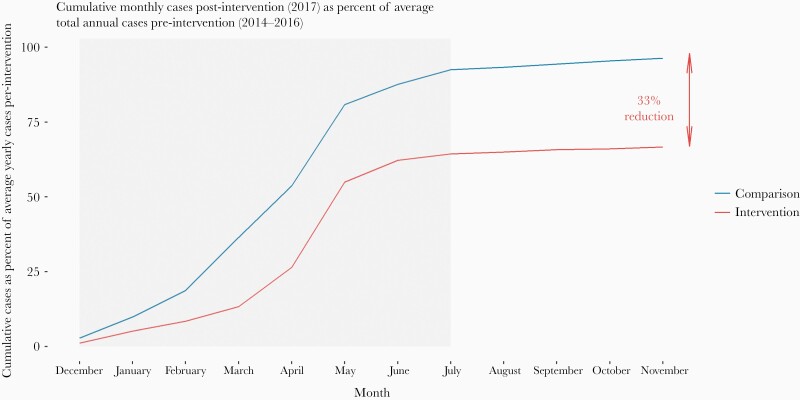
Cumulative monthly cases postintervention as percentage of average number of cases from the previous 3 seasons, by programmatic mass drug administration and comparison locations. For example, by November, only 67% of cases we would have expected in the entire season (based on the average from previous seasons) have occurred. The gray box indicates the duration of the malaria season.

Percentage change in incidence after the start of the interventions was heterogeneous within both groups (Figure 3). Of the 10 facilities in the pMDA group, only Itapa Health Post saw an increase in incidence (39% increase) between the baseline and postintervention periods, in part due to a very low incidence in the 2015/2016 season. All others saw decreases (maximum 89%). In the comparison group, 5 HFCAs saw an increase (maximum 50%) and 15 saw a decrease (maximum 88%). There was an overall reduction of 33% in cumulative cases during the postintervention period in the pMDA group compared with the average from the 3 previous seasons (Figure 4). In the comparison group, there was only a 4% reduction in cumulative areas compared with previous seasons.

## DISCUSSION

We evaluated whether there was a difference in the overall level and the trend in confirmed malaria case incidence after pMDA in selected HFCAs in Southern Province as opposed to a comparison group that received no pMDA. We found that there was an overall 46% larger decrease in confirmed malaria case incidence in the period immediately after the intervention in the pMDA group, in addition to the decrease observed in the comparison group, but no significant trend change. These results are very similar to the incidence results reported in the Zambia MDA CRCT, which found a 37% percent incidence decline in its low transmission strata [14]. However, most pMDA HFCAs still experienced a peak in incidence during the 2016–2017 transmission season (Supplementary Figure 1), even though the cumulative incidence was lower. In addition, the comparison of the cumulative incidence between the postintervention season and the preintervention seasons suggests that pMDA caused an immediate reduction in case incidence lasting until approximately 2 months after the second pMDA round, likely due to the prophylactic effect of DHAP [15].

The resurgence in incidence a few months after pMDA prompts the questions of whether 3 pMDA rounds would delay the seasonal spike enough that it would never occur, what difference improved vector control might make in preventing the resurgence at the end of the season, and what impact travel from other areas may have. Recently, several related MDA studies in the Mekong area in southeast Asia have conducted 3 rounds separated by only 1 month, and they showed a range of outcomes, from a time-limited reduction in incidence in settings with much lower transmission than the areas included in our analysis [16, 17] to near-cessation of *P falciparum* clinical incidence 1 year after implementation in a setting with comparable incidence [18].

The peak in transmission also coincides with waning efficacy of IRS (IRS with Actellic was conducted in November and December of 2016, and it is estimated to be effective for approximately 4 to 5 months in Zambia [19]). Although it is recommended that MDA only be conducted in areas with high vector control and community case management coverage, not all HFCAs received IRS. Therefore, improved coverage and duration of vector control interventions may lead to a larger MDA effect. Deploying mosquitocidal drugs, such as ivermectin, in conjunction with DHAP is another future option to increase impact. Regarding travel from other areas, an analysis after the original CRCT found that travel likely had very little impact on the effectiveness of MDA in Southern Province [20].

There are several implementation lessons for future MDA interventions. Most important is ensuring that supply procurement and delivery happen well in advance of implementation, especially in places prone to flooding or other mobility disruptions. Such disruption likely caused the reduced coverage in the second round (57% vs 70% coverage) and potentially led to lower impact. Robust sensitization programs are also essential to success. In addition, new digital tools that allow health workers to locate households and mark their status individually are already being used for IRS in Zambia and are promising for MDA. Finally, programs should also consider resistance monitoring if conducting MDA over the course of multiple years.

This study was limited due to lack of availability of long-term follow-up data. Our analysis shows that MDA was implemented programmatically in Zambia with results similar to those under experimental conditions, but it was not able to address how well those gains are maintained in the long term. Better data about concurrent interventions would have also allowed us to more accurately control for external factors when determining the impact of pMDA.

Interrupted time series are a promising tool for evaluating impact [13]. However, the overall drop that we observed in both the control and comparison groups highlights the critical need for interrupted time series analyses to have an appropriate comparison group. Without our comparison group, we may have overestimated the effect of the intervention.

In the past, evaluations using routine data have often been challenging due to lack of data collection or poor-quality data. In the MDA literature in particular, only 2 recently published studies used routine data, including a study in Sierra Leone where the data had to be digitized specially for use in the analysis [21, 22]. Analyzing the impact of programmatic interventions using routine surveillance data has many limitations. However, understanding and quantifying what is currently happening is of huge importance. Conducting cluster-randomized trials to estimate the impact of every intervention combination in every transmission setting is entirely unfeasible. In addition, estimating the effectiveness of interventions that are programmatically implemented is needed. Analyses such as the one presented here should be conducted routinely by national malaria control programs to better guide their strategy and decision making.

## CONCLUSIONS

Interrupted times series with comparison group results suggest that 2 rounds of pMDA with DHAP, implemented during 1 year with moderate coverage in a low transmission area with varying levels of vector control, had a significant short-term effect on confirmed malaria case incidence. Programmatic MDA can help temporarily decrease malaria transmission in this type of setting. However, as was true in trial conditions, it was insufficient to reach elimination. Additional tools, such as digital systems that allow better tracking of interventions, better coverage of vector control, and reactive interventions, such as reactive IRS or reactive fMDA, should be explored to achieve elimination. Quality routine data not only give us a timely picture of what is happening in a country but also create the opportunity to evaluate programmatic interventions so that we can understand whether policy or delivery strategies need to be adapted or changed.

## Supplementary Data

Supplementary materials are available at *The Journal of Infectious Diseases* online. Consisting of data provided by the authors to benefit the reader, the posted materials are not copyedited and are the sole responsibility of the authors, so questions or comments should be addressed to the corresponding author.

**Supplementary Figure 1.** Incidence patterns and model fit in individual health facility catchment areas. Vertical lines show the time of intervention. Note change in y scale between facilities.

**Supplementary Table 1.** This table shows a data schematic for the variables used in the ITSc model to aid in understanding its structure. Each row represents values in the data for a generic HFCA in either the intervention or comparison group. Each column represents a different point time. The vertical line in the middle indicates when the first round of pMDA was conducted. For example, the “months since intervention” variable is 0 for both groups before the point of intervention, and then it increases by 1 each month after. The value of “the interaction between months since beginning of study and intervention group” increases by 1 each month for the intervention group, but remains 0 for all time points for comparison HFCAs.

**Supplementary Table 2.** Covariate summaries by season year (November–October) during the preintervention and postintervention periods in the pMDA (10 HFCAs) and comparison (20 HFCAs) groups.

jiaa434_suppl_supp_Supplementary_Table_1

jiaa434_suppl_supp_Supplementary_Table_2

jiaa434_suppl_supp_Supplementary_Figure_1

## Notes

***Acknowledgments.*** We thank the Zambian National Malaria Elimination Centre for support in this analysis and the generous sharing of routine and intervention data that they provided. In addition, this analysis would not have been possible without the district, health facility, and community health workers involved in the programmatic mass drug administration and routine data collection. We also thank Helena Martí (Barcelona Institute for Global Health [ISGlobal]) for advice on dealing with seasonality and other statistical issues and Ruth Ashton (Tulane University) for advice about lessons learned in interrupted time series analyses. Futhermore, we thank Manny Lewis for copyediting the manuscript. Finally, we thank the Bill & Melinda Gates Foundation for their financial support to the Malaria Control and Elimination Partnership in Africa project, which made this analysis financially possible.

***Author contributions.*** All authors had access to the data in the study and reviewed the manuscript.

***Disclaimer.*** The funding source had no role in the conduct, analysis, or interpretation of results of the study.

***Financial support.*** This analysis was funded by a grant from the Bill & Melinda Gates Foundation (OPP1134518 Elimination Scale Up in Zambia).

***Potential conflicts of interest.*** All authors: No reported conflicts of interest. All authors have submitted the ICMJE Form for Disclosure of Potential Conflicts of Interest.

Presented in part: American Society for Tropical Medicine and Hygiene Conference, October 28–November 1, 2018, New Orleans, LA; The Malaria Endgame: Innovation in Therapeutics, Vector Control, and Public Health Tools Keystone Symposia Conference, October 30–Novemeber 2, 2019, Addis Ababa, Ethiopia.
