# Supplementary material for: Evaluating the Impact of Programmatic Mass Drug Administration for Malaria in Zambia Using Routine Incidence Data
Source: J Infect Dis. 2020 Jul 21;225(8):1415–23. doi: 10.1093/infdis/jiaa434 (PMC9016426; doi:10.1093/infdis/jiaa434)
Supplement: jiaa434_suppl_supp_Supplementary_Table_1 [file jiaa434_suppl_supp_supplementary_table_1.docx]

| **Variable** | **Group** | **Month 1** | **Month 2** | **Month 3** | **Month N** | **Month N+1** | **Month N+2** |
| --- | --- | --- | --- | --- | --- | --- | --- |
| Interaction between pre/postintervention time period and intervention group (level change) | Intervention | 0 | 0 | 0 | 1 | 1 | 1 |
|  | Comparison | 0 | 0 | 0 | 0 | 0 | 0 |
| "Interaction between months since intervention and intervention group (slope change)" | Intervention | 0 | 0 | 0 | 1 | 2 | 3 |
|  | Comparison | 0 | 0 | 0 | 0 | 0 | 0 |
| "Interaction between months since beginning of study and intervention group" | Intervention | 1 | 2 | 3 | N | N+1 | N+2 |
|  | Comparison | 0 | 0 | 0 | 0 | 0 | 0 |
| Months since intervention | Intervention | 0 | 0 | 0 | 1 | 2 | 3 |
|  | Comparison | 0 | 0 | 0 | 1 | 2 | 3 |
| Months since start | Intervention | 1 | 2 | 3 | N | N+1 | N+2 |
|  | Comparison | 1 | 2 | 3 | N | N+1 | N+3 |
| Pre/post intervention (binary) | Intervention | 0 | 0 | 0 | 1 | 1 | 1 |
|  | Comparison | 0 | 0 | 0 | 1 | 1 | 1 |
| Intervention group (binary) | Intervention | 1 | 1 | 1 | 1 | 1 | 1 |
|  | Comparison | 0 | 0 | 0 | 0 | 0 | 0 |
|  |  |  |  |  |  |  |  |
|  |  |  |  |  | **<- First round of pMDA** | | |

Supplemental Table 1: This table shows a data schematic for the variables used in the ITSc analysis to aid in understanding its structure. Each row represents values in the data for a generic facility in either the intervention or comparison. Each column represents a different point in time. The line in the middle indicates when the first round of pMDA was conducted. For example, the “months since intervention” variable is 0 for both groups before the point of intervention, and then increases by one each month after. The value of “Interaction between months since beginning of study and intervention group” increases by one each month for the intervention group, but remains 0 for all time points for comparison facilities.
