# Supplementary material for: Evaluating the Impact of Programmatic Mass Drug Administration for Malaria in Zambia Using Routine Incidence Data
Source: J Infect Dis. 2020 Jul 21;225(8):1415–23. doi: 10.1093/infdis/jiaa434 (PMC9016426; doi:10.1093/infdis/jiaa434)
Supplement: jiaa434_suppl_supp_Supplementary_Table_2 [file jiaa434_suppl_supp_supplementary_table_2.docx]

| **Variable** | **Group** | **Year** | | | |
| --- | --- | --- | --- | --- | --- |
|  |  | **2014** | **2015** | **2016** | **2017** |
| Elevation (m) | Intervention | 1009 | 1009 | 1009 | 1009 |
|  | Comparison | 1033 | 1033 | 1033 | 1033 |
| Estimated Population by health facility catchment (average) | Intervention | 3720 | 3752 | 3859 | 3978 |
|  | Comparison | 4153 | 4226 | 4340 | 4408 |
| Average monthly NDVI | Intervention | 151.24 | 149.60 | 148.47 | 151.17 |
|  | Comparison | 147.87 | 147.49 | 146.09 | 148.16 |
| Average Monthly Rainfall (mm) | Intervention | 71 | 64 | 56 | 80 |
|  | Comparison | 68 | 64 | 55 | 77 |
| Number of HFCAs receiving IRS | Intervention | 0 | 8 | 5 | 8 |
|  | Comparison | 0 | 18 | 4 | 7 |
| Estimated IRS Coverage at Time of Spraying (sprayed HFCAs only) | Intervention | None sprayed | 30% | 50% | 87% |
|  | Comparison | None sprayed | 21% | 75% | 54% |
| Average Estimated Usable Nets per Person (distribution at the end of 2014) | Intervention | 0.36 | 0.60 | 0.40 | 0.27 |
|  | Comparison | 0.34 | 0.64 | 0.43 | 0.28 |
| Average Monthly Non-Malaria OPD Attendance per Person | Intervention | 0.13 | 0.15 | 0.15 | 0.14 |
|  | Comparison | 0.25 | 0.23 | 0.21 | 0.21 |
| Percent of Malaria Cases Treated by CHW | Intervention | 28% | 24% | 21% | 23% |
|  | Comparison | 43% | 43% | 40% | 42% |
| Tests per Index Case Conducted during Reactive Case Detection | Intervention | 3.46 | 2.54 | 2.87 | 4.91 |
|  | Comparison | 4.71 | 5.28 | 4.33 | 5.65 |
