# Supplementary figures and images for: Evaluating the Impact of Programmatic Mass Drug Administration for Malaria in Zambia Using Routine Incidence Data
Source: J Infect Dis. 2020 Jul 21;225(8):1415–23. doi: 10.1093/infdis/jiaa434 (PMC9016426; doi:10.1093/infdis/jiaa434)

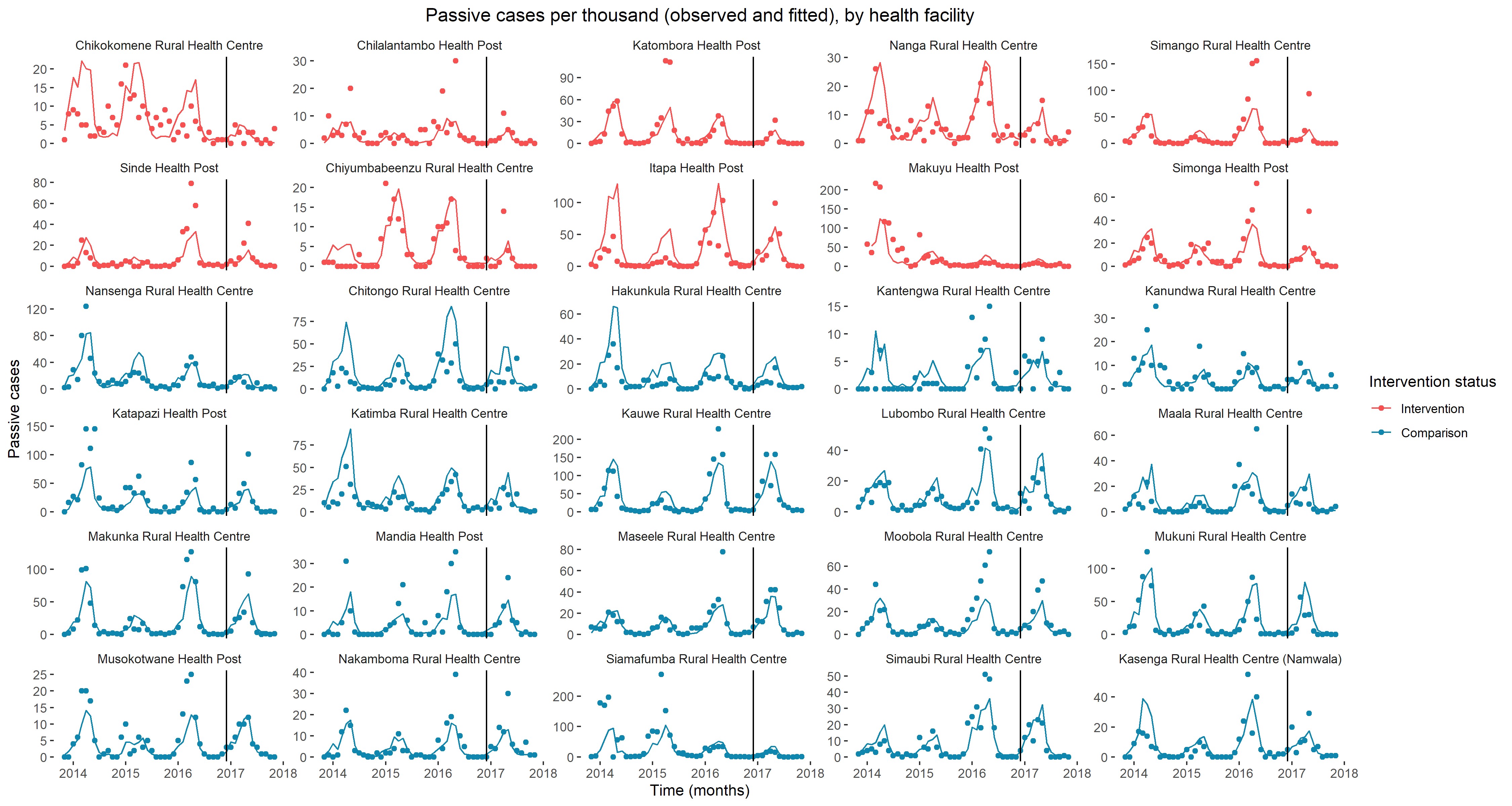

Supplement: jiaa434_suppl_supp_Supplementary_Figure_1 [file jiaa434_suppl_supp_supplementary_figure_1.jpeg]
